# A Detailed Study on the Public and Health Worker Perception and Level of Knowledge on the Topic of Radiation and Nuclear Energy in Oman

**DOI:** 10.3390/ijerph23060755

**Published:** 2026-06-04

**Authors:** Hajir Al Hamrashdi, Ismail Al Yahmadi, Fatma Al Ma’mari

**Affiliations:** Department of Physics, Sultan Qaboos University, Muscat P.O. Box 36, Oman

**Keywords:** radiation, misinformation, knowledge

## Abstract

**Highlights:**

**Public health relevance—How does this work relate to a public health issue?**

- Surveying the public on their knowledge on the topic of radiation and nuclear applications is directly tied to public health as it measures readiness of individuals and communities to real health risks.
- Surveying the public on their knowledge on the topic of radiation and nuclear applications is directly linked to public health as it shapes how individuals and communities respond to medical care and emergencies.

**Public health significance—Why is this work of significance to public health?**

- First, public understanding affects health behaviours. To start with, if people overestimate the risk of radiation and nuclear applications, such as diagnostic X-ray, they may avoid the procedure, and the quality of healthcare will be compromised. Vice versa, if the risk from radiation and nuclear applications is underestimated, unnecessary doses might be received by patients.
- Understanding the gaps in the set of facts on this topic is crucial for emergency preparation, especially at times like the ones we experience now. Indeed, public response to emergencies, based on their level of knowledge, of course, can make a crisis worse or mitigate its impact. Subsequently, it can either reduce the associated health aftermath of the incident or amplify it.

**Public health implications—What are the key implications or messages for practitioners, policy-makers and/or researchers in public health?**

- Measuring public health knowledge on the topic of radiation and nuclear applications will help authorities identify the gaps in knowledge and directly assess them in designing educational programs informed decisions and clarify any misunderstanding.
- Radiation is often linked to danger and fear; hence, assessing public knowledge will directly help health agencies to tailor messages that are clear, credible and culturally appropriate.

**Abstract:**

Background radiation and nuclear applications are deeply embedded in modern life. With the continuous advancement in nuclear techniques, daily radiation exposure is commonplace. The level of knowledge within this field among the general public must therefore be assessed to ensure that fundamental and essential information on radiation and nuclear energy is attained and to prevent the spread of misinformation. The aim of this study is to evaluate the level of knowledge and awareness of this topic among the general public in Oman. The study was performed using a cross-sectional survey specifically designed to measure the knowledge of different aspects in the field of radiation and nuclear applications. We achieved this aim by assessing the level of knowledge among the general public and medical professionals. Overall, 500 individuals participated in the study, 75 of whom were from the medical field. The results demonstrate varying levels of knowledge on this topic. In general, the public and medical professionals show a high level of confidence in basic information; however, the level of knowledge and confidence decreases to intermediate and, in some cases, low levels with questions at fundamental and intermediate levels of knowledge. This decrease was clearly observed among topics related to the nature of radiation, the origin of radiation, radiation protection, and radiation applications. Our findings demonstrate conclusively that the public and medical professionals in Oman have limited knowledge in this field; therefore, appropriate measures must be implemented.

## 1. Introduction

The topic of radiation and nuclear applications consistently emerges as a highly controversial issue across media platforms. Amid ongoing political tensions worldwide, radiation and nuclear applications are frequently portrayed through negative framing and misleading interpretations communicated by both specialists and non-specialists. Research demonstrates that media coverage of radiation and nuclear applications is commonly characterized by intense, short-term, biased dissemination of information [1]. The media, in all of its forms, contributes to shaping public knowledge of radiation and nuclear applications; in most cases, however, this source of knowledge is inaccurate and lacks scientific grounding [2,3,4].

It is well established that ionizing radiation is a fundamental component of nature to which humans are exposed on a daily basis. Advances in ionizing radiation technologies have contributed to increased levels of radiation exposure. Within the medical field, for example, the number of referrals for radiological examinations has risen significantly. Reports indicate that radiation doses received by the public from medical procedures are now comparable to those from natural background radiation [5,6,7]. Despite this alarming increase, the dissemination of accurate information regarding ionizing radiation to patients and the general public remains limited [7,8,9]. This lack of effective communication represents an additional contributing factor to misunderstanding and misinformation in the field of radiation and nuclear applications.

A lack of comprehensive knowledge, combined with misleading or inaccurate information on a sensitive topic such as this, can lead to unfavourable outcomes, including false judgments, exaggerated interpretation of events, unjustified fear and, in some extreme cases, public panic. In addition, the sensitive nature of the topic, particularly in relation to risk assessment, requires a well-founded understanding. However, the topic remains inherently broad and conceptually complex. There is thus a pressing need to address this knowledge gap and to shift the focus from short-term, inaccurate dissemination of information toward a sustainable, concept-based educational approach capable of accurately communicating information on background radiation and nuclear applications. Despite the small number of published works in this field, an increase in peer-reviewed studies has been reported in the literature over the past two decades. The authors of existing studies in the field have primarily focused on assessing or evaluating the level of knowledge among different societal groups, including medical professionals and the general public [4,7,10,11,12,13,14,15,16]. Nevertheless, the literature highlights an evident gap in research related to communication on radiation and nuclear applications worldwide, particularly in the Middle East [1].

In this study, we aim to evaluate the public’s knowledge in Oman regarding radiation and nuclear applications through a cross-sectional survey. The study is structured to progressively assess participants’ understanding, beginning with questions that examine basic general knowledge of the topic. Thereafter, we focus on members of the public with prior preliminary knowledge on the topic of radiation sources and radiation protection. In the final stage of the evaluation, we aim to assess knowledge among medical professionals regarding different radiation-based medical technologies. This approach will form a systematic review of public and medical professionals’ knowledge of this field in Oman. It is anticipated that the findings of this study will support educational reform, patient awareness, and preparedness for radiation-related emergencies, particularly in light of rising tensions in the region.

## 2. Materials and Methods

A multiple-choice, cross-sectional survey was used to conduct this study. The survey comprised four sections and a total of 29 questions (the full survey is available in Appendix A). The first section collected demographic information, including age, gender, educational background, and profession. The second section consisted of nine questions designed to assess participants’ general knowledge of radiation, including knowledge commonly acquired through everyday experiences and habits, such as watching documentaries or following public news. Responses from this section were subsequently used to classify the participants into two groups: those with prior educational knowledge in the field and those with none. The third section comprised 11 questions. The final section was specifically dedicated to workers in the medical field due to the importance and sensitivity of this group within wider society. Additionally, the background knowledge of healthcare professionals in this field is pivotal, as they are most likely to encounter radiation-related practices at some stage during their careers. Moreover, medical professionals represent a substantial percentage of the total population. As of the latest census, Oman has a population of 3,056,407. According to the annual health report published by the Ministry of Health in Oman, the density of medical professionals is 65 per 10,000 population [17]. Information from the same report indicates that 71,180 individuals work in the medical field in Oman. The written survey was initially reviewed and approved by Sultan Qaboos University (the Principal Investigator’s institute) survey unit. Subsequently, approval was sought to conduct the survey at a national level. The survey was comprehensively assessed and approved by the National Centre of Statistics and Information in Oman. Approval number 2025-1190 was granted on 20 February 2025, and the research team was given six months to conduct the survey nationwide. The full survey can be found in the appendices.

The survey was written using Google Forms (Google LLC, Mountain View, CA, USA) and distributed electronically among the general public. Multiple digital platforms were utilized to distribute the survey. This measure was taken to ensure that the study reached the greatest possible number of participants. The platforms via which the survey was distributed included Gmail from Google (Google LLC, Mountain View, CA, USA), WhatsApp and Instagram from Meta (Meta Platforms, Inc., Menlo Park, CA, USA), and SMS messaging. These platforms were used due to their high level of popularity and widespread use among the public in Oman. Both randomized and snowball sampling techniques were used to increase the number of participants and include the broadest possible representation of the public. 

## 3. Results

**Figure 1:**
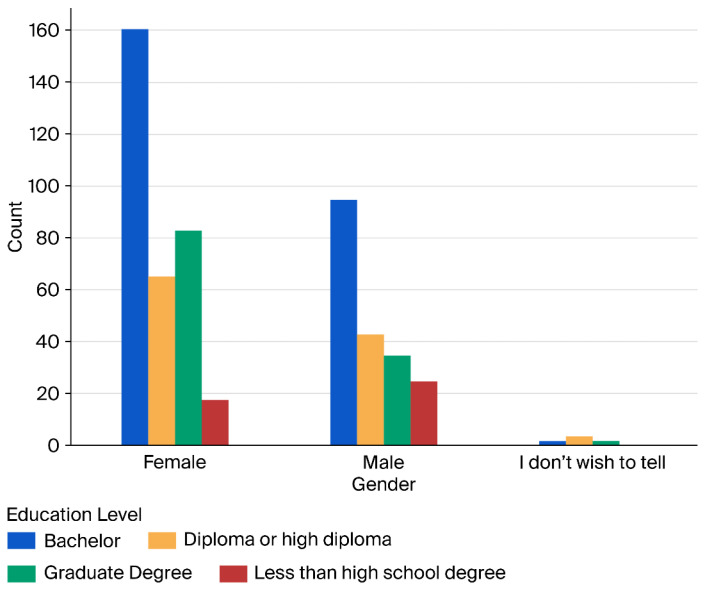
Education level distribution across genders.

A total of 500 responses were obtained from the general public. The highest response rate was observed in the 18–44 age group, with the 35–44 age category contributing to the largest number of responses. More complete responses were obtained from female participants, with 60.2% of total responses coming from females compared to 38.4% from males, with only 1.4% not wanting to disclose their gender. In terms of educational attainment, the majority of respondents held a bachelor’s degree or higher (70.0%), with 30% holding a diploma, higher diploma, or lower level of education. The distribution of education by gender is shown in Figure 1. As shown in the figure, the largest respondent group consisted of bachelor’s degree holders, followed by those holding a diploma or high diploma, a graduate degree, and lastly a high school education or lower.

The focus of the survey then shifted to general and basic knowledge of radiation. In the first question, participants were asked whether they believe radiation actually exists, to which over 98.0% responded “yes”. In response to the second question, over 91.0% of participants believe that radiation is a form of energy, while only 2.2% believe that it is not, and 6.6% responded with “I don’t know”. In the following question, participants were asked whether radiation can be seen and touched, to which the responses indicated that participants possessed less sound knowledge, with 78.8% answering correctly, 12.0% responding that it can be seen and touched, and 9.2% stating that they do not know the answer to the question. The responses to the question of whether radiation is a single type were as follows: 89.0% believe that there is more than one type of radiation, while only 1.0% believe that there is only one type of radiation, and 9.0% responded with “I don’t know”. The subsequent question was as follows: “Do humans and living creatures receive radiation externally only?” The responses were as follows: 91.0% answered “yes”, 2.0% answered “no”, and 7.0% answered “I don’t know”. This finding clearly demonstrates that the public has limited knowledge of the methods through which radiation can be received. The public perspective on radiation was evaluated with the following statement: “Radiation is always harmful and can’t be useful”. To this question, 85.0% answered “no”, 7.0% answered “yes”, and 8.0% responded with “I don’t know”. The responses indicated that the public are aware of the different uses of radiation. The following question yielded different responses, with 61.0% believing that radiation is not contagious, 19.0% believing that radiation is contagious, and 21.0% stating that they do not know the answer to the question. The statement “Naturally occurring radioactive material, also known as NORM, can be found anywhere around the World” generated the most indecisive responses among participants, with only 56.0% answering the question correctly with “yes”, 24.0% answering “no”, and 20.0% responding with “I don’t know”. The final question in the general knowledge category reflected a relatively strong level of knowledge among the general public, with 82.0% agreeing that radiation is part of our daily lives and responding “yes”, 8.0% responding “no”, and 10.0% responding with “I don’t know”.

**Figure 2:**
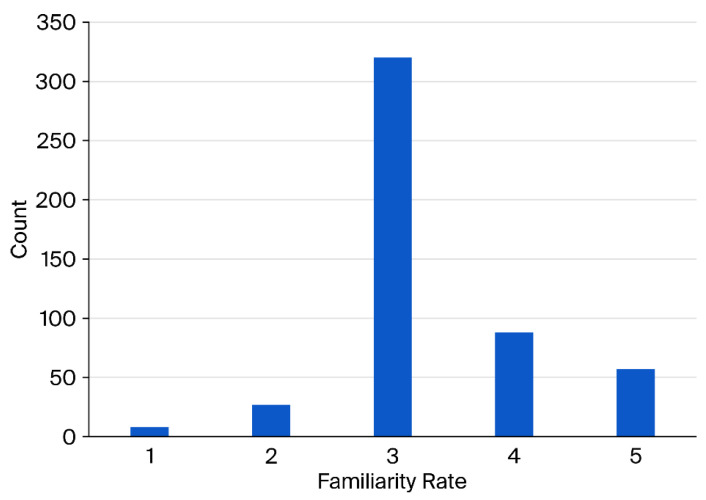
The familiarity of participants with the topic of radiation (5 = very familiar, 4 = familiar, 3 = average familiarity, 2 = not very familiar, and 1 = not familiar).

In the subsequent section, we assessed intermediate knowledge of radiation and nuclear applications. We began by categorizing the respondents into two groups: those with prior educational knowledge of radiation and those with none. Of the respondents, 57.8% indicated that they have prior knowledge, while 42.2% indicated that they do not. Information on their familiarity with the concept of radiation is shown in Figure 2, where the x-axis represents their degree of familiarity with the topic (5 represents great familiarity and 1 represents no familiarity).

The first question that explored intermediate knowledge of radiation was as follows: “Which of the following types of radiation are you aware of? (Select all that apply)”. Options included Alpha particles, Beta particles, Gamma rays, X-rays, Neutron radiation, and None of the above. Of the participants, 23.0% are familiar with all five listed types of radiation, 11.2% are familiar with four out of the five types, and 11.2% are familiar with three out of the five types. Of the negative responses, 19.2% indicated that they are familiar with only one or two types of radiation, and 42.2% indicated that they are not familiar with any of the listed types of radiation, reflecting the number of respondents who also indicated they have no prior knowledge of radiation. 

The second question was as follows: “Where does natural radiation come from? (Select all that apply)”. This question was employed to assess the public’s understanding and awareness of natural background radiation. Answers included Cosmic rays, Radon gas, Soil and rocks, Medical X-rays, and Nuclear power plants. Only 7.4% chose all correct answers, with 30.2% choosing at least one correct answer. These percentages reflect poor knowledge of this topic. To measure the public understanding of medical-related practices that involve radiation, the following question was included: “What are the common uses of radiation in medicine?” The participants were given the following options: X-ray imaging, CT scans, Radiation therapy for cancer, MRI scans, and Ultrasound imaging. Of the respondents, 15.2% chose all options, 25.0% chose only the correct options, and 47.2% chose at least one incorrect option. The findings suggest that while a minority of respondents demonstrated accurate knowledge regarding radiation in medicine, a higher percentage selected at least one incorrect option, thus once again indicating the presence of misconceptions and gaps in the understanding of the uses of radiation in medicine.

### 3.1. Radiation Safety

The first question in the radiation safety section was as follows: “Which of the following is a common safety measure when working with radiation?” The responses reflected substantial background knowledge, with 82.0% of those with previous knowledge of radiation answering the question correctly. The high percentage of positive responses in this category was observed for the following question, with 91.6% responding “yes”: “Can high levels of radiation be harmful to health?” Of the remaining respondents, 4.8% answered “no”, and 3.5% responded, “I don’t know”. The subsequent question was employed to assess the participants’ views and perspectives on the topic of radiation safety. The question asked was as follows: “Have you ever been concerned with radiation exposure?” In response, 65.7% answered “yes”, 18.7% answered “no”, and 15.6% answered “I don’t know”. Although individuals who are concerned about radiation represent the highest percentage, covering almost 35.0% of the sample, answering otherwise triggers the curiosity of the authors and the need to improve such a question in the future to include an open-ended part to the question.

### 3.2. Media, Education, and Awareness

**Figure 3:**
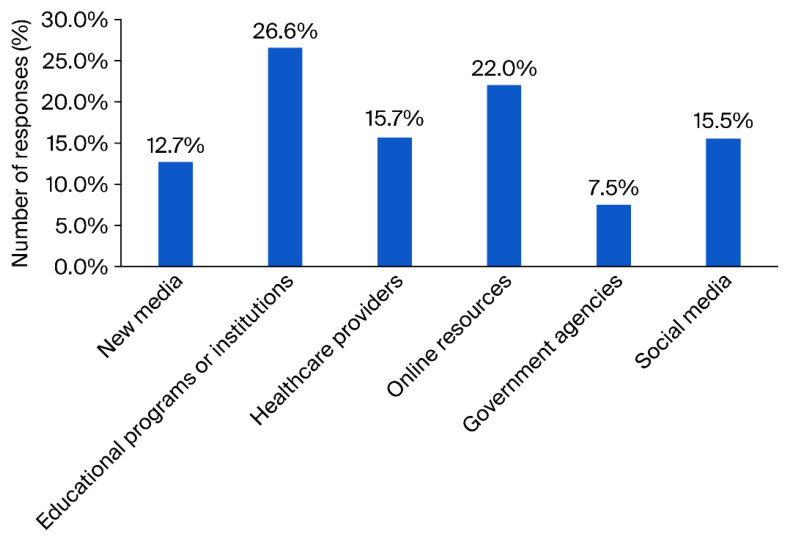
Public opinion on sources of information about radiation and its effects.

The impact and credibility of information and news in the field of nuclear radiation are undeniable. As such, we surveyed the main sources upon which the participants rely to gain conceptual knowledge, including breaking news and analytical descriptions of events. The first question was as follows: “Where do you usually obtain information about radiation and its effects? (Select all that apply)”. Figure 3 illustrates the options given to the participants and their responses. The results demonstrate that most individuals rely on educational programs and online resources to learn about radiation—an expected result, yet one that demonstrates the importance of enriching and monitoring these two learning outlets. Next, participants were asked to give their opinion on two matters: First, whether they would be interested in learning more about radiation safety and its effects and second, whether they think radiation education is important. Of the respondents, 77.2% answered “yes” to the first question, 7.3% answered “no” to the question, and 5.6% responded with “maybe”. To the second question, 90.3% responded with “yes”, 1.0% responded “no”, and 8.7% responded “maybe”. The responses reflect general positivity toward learning more about radiation. Another important aspect in positive and credible awareness was addressed by asking the participants about their opinion on the role of universities and higher education in promoting accurate awareness of radiation knowledge. Of the respondents, 87.9% agree with the importance of promoting awareness in higher education, 3.8% do not think it is important, and 8.3% are neutral. This pronounced positive attitude toward promoting radiation awareness reflects a strong acknowledgement of the need to provide a platform for enhancing radiation literacy and addressing misconceptions related to radiation.

### 3.3. Medical Professionals

**Figure 4:**
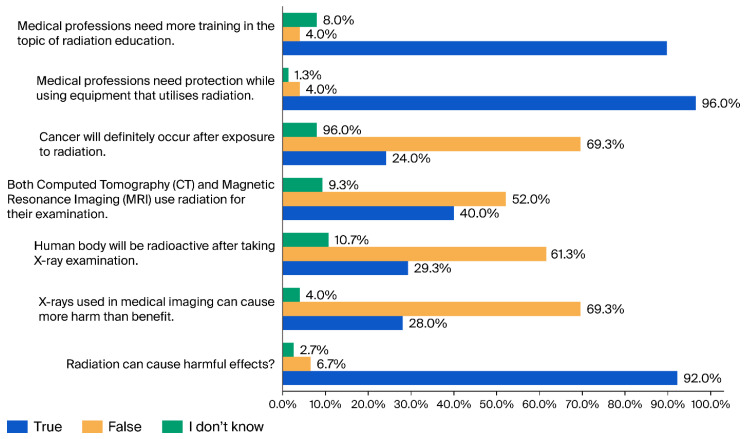
Medical professionals’ responses to the survey questions related to radiation knowledge in the medical field.

The final part of the survey focused on medical professionals, with only 15.0% of the total participants indicating that they work in the medical field. The responses of the participants to the questions are illustrated in Figure 4 (the order of questions as they were asked is shown from bottom to top). The response to the first question was overwhelmingly biased toward believing that radiation can cause harmful effects, thus demonstrating that medical professionals are fully aware of the negative effects of radiation in medicine. The response to the second question reflects the sound background knowledge of the majority of participants from the medical field on the benefits versus risks of diagnostic X-ray. Participants were required to respond to the following statement: “The human body will be radioactive after undergoing an X-ray examination”. Surprisingly, and an output that is not aligned with previous responses, 29.3% believe this statement to be true; in comparison, 61.3% believe it to be false, and 10.7% state that they do not know. This finding highlights a serious issue that can affect patient experience in healthcare facilities. Medical professionals were then asked to respond to the following statement: “Both computed tomography (CT) and magnetic resonance imaging (MRI) use radiation for diagnostic imaging”. This question assesses whether participants possess a deeper understanding of radiation types and the difference between two of the most widely used diagnostic technologies. As can be seen in Figure 4, the responses were close, with 40.0% answering “true” and 52.0% answering “false”. This finding is a clear indication that misconceptions need to be addressed among medical professionals. The participants were then asked to respond to the following statement: “Cancer will definitely occur after exposure to radiation”. This question again assesses whether participants possess a deeper understanding of the negative side effects of radiation. The results were as follows: 24.0% answered incorrectly with true, 69.3% responded with false, and 8.0% responded with “I don’t know”. Medical professionals’ basic knowledge on radiation protection measures was evaluated with the following statement: “Medical professionals need protection while using equipment that utilizes radiation”. The responses were overwhelmingly positive, with 96.0% answering “true”, 4.0% answering “false”, and 1.3% answering with “I don’t know”. Lastly, medical professionals were asked to give their opinion on whether medical professionals require more training on the topic of radiation. The results presented in Figure 4 clearly demonstrate that the majority of medical professionals believe that more training is required in this field, with only 4.0% believing that training is not required.

## 4. Discussion

Demographic data were similar to those found in other studies, with the early middle age group being the most represented age group and female participants demonstrating a higher response rate than males [10,15,16].

The responses to the basic radiation knowledge section indicated that, in general, the public has an appropriate level of basic knowledge on this topic. Questions 1 to 6 were answered with a correctness percentage higher than 75%, a descriptive indication that reflects the sample proportion of correctness. Applying a one-tailed proportion z-test for questions 1 to 6 demonstrates that the population correctness exceeds 75% for every question with a 5% confidence level. The correctness level drops significantly below 75% for questions 7 and 8. The two questions reflect a slightly higher level of basic knowledge on the topic, serving as a useful indicator of the areas within the basic knowledge category that need to be addressed more thoroughly, in order to reduce misconceptions among the public. The final question in the general knowledge section addressed the topic of general awareness and humans’ daily experience of background radiation. The responses were overwhelmingly correct with a 95% confidence level.

Within the third part of the survey, knowledge that is more likely to be acquired through a structured educational system was assessed. Of the respondents, 57.8% indicated that they have prior knowledge of radiation science. The series of questions started with a direct question of how familiar they were with the topic of radiation, with 7.0% indicating that they are not particularly familiar with the topic, 64.0% indicating that they have medium confidence or familiarity with the topic, and 24.0% indicating that they have high confidence in their knowledge, i.e., a quarter of the total sample. The first question that assessed the public’s in-depth knowledge was on the different types of ionizing radiation. Only 23.0% correctly identified all of the listed radiation fields. The score correlates with the level of familiarity recorded by participants. The second question in this category was used to investigate public knowledge of the sources of natural background radiation. Only 7.4% chose all correct answers, and 30.2% chose at least one correct answer. A clear gap in public knowledge was identified regarding this core aspect of understanding natural background radiation. The following question focused on man-made applications of radiation in the medical field. Of the respondents, 24.0% chose the correct answer. The percentage once again correlates with those who claimed they have high confidence in their knowledge.

The fundamental introductory level knowledge on the topic of radiation safety was assessed by rating the correctness of the responses. The response of the participants to the first question on radiation safety reflected satisfactory and an in-depth background knowledge of safety measures when addressing ionizing radiation, with a significant 82.0%. The strong foundation of the public in the topic of radiation safety continued to show in the following question, with 91.6% responding “yes” to “Can high levels of radiation be harmful to health?” This finding can be attributed to numerous factors. The authors favour the following interpretation: the high impact of nuclear accidents on the news and in the media, the emphasis in the literature on such events, and the strong focus on health side effects whenever incidents occur, together with the association of radiation with carcinogenic diseases [18], have collectively contributed to greater public awareness of this particular aspect of radiation and nuclear applications.

Sources of knowledge and information were explored in the following section. The first question in this section asked which sources of information the participants prefer. The sources, listed in order of popularity, were as follows: educational programs, online resources, healthcare providers, social media, news media, and, lastly, government agencies. The number of responses per source shows that there is no substantial preference for one source of information over another, except for government agencies, as a source of information, with only 7.5% of the participants considering it as a source of information. The responses to this question offer a critical insight into the public sources of information on this topic and subsequently which sectors of information the government should target and focus on to enhance knowledge among the public in this field. The participants were then asked whether they were interested in learning more about radiation safety and radiation effects. The results demonstrated that the majority are interested in learning more—an outcome that indicates the need and demand for more investment in educating the public on matters in this field. This outcome was substantiated in the responses to the following questions, with over 90.0% of the participants responding “yes” to the question “Do you think radiation education is important?” Over 80.0% responded “yes” to the question “Do you think universities need to promote the accurate awareness of radiation knowledge?” Overall, no clear correlation between educational level and knowledge in the field of radiation and nuclear applications was found.

Medical professionals’ responses were central to this survey. The fact that healthcare providers are ranked third as a source of knowledge by the public and the criticality of their roles made them a crucial demographic subgroup in the study. Overall, 15.0% of the participants stated that they work in the medical field. The question in this section of the survey focused on evaluating knowledge among medical professionals on the medical applications of ionizing radiation. In the first question, the participants were asked an introductory question regarding whether they believe radiation can cause harmful effects. The responses indicated a high level of knowledge on this topic, with 92.0% answering the question correctly. However, one surprising observation was the fact that, in the following question, fewer than 70.0% of the participants from this subgroup do not believe X-rays used in medical imaging can cause more harm than benefit. The following question in this part of the survey addresses knowledge among medical professionals regarding the contagion or the passing of radiation effects after a routine radiation examination. The responses suggested that medical professionals have moderate knowledge of this topic, with less than 70.0% answering the question correctly. The level of knowledge was closer to low in the question that follows, with 52.0% of the medical professionals believing that both computer tomography (CT) and magnetic resonance imaging (MRI) involve the use of ionizing radiation. Medical professionals were then asked if they believe that cancer will occur after radiation exposure, with the responses suggesting, once again, an intermediate level of knowledge regarding this topic. Overall, the responses from medical professionals demonstrate an intermediate level of knowledge regarding the fundamental concepts related to the use of ionizing radiation in the medical field. As part of the last two questions, medical professionals were invited to give their opinion on two important topics. The first one was as follows: “Do you think medical professionals need protection while using equipment that utilizes radiation?” The majority of respondents answered yes. The last question was as follows: “Do you think medical professionals need more training on the topic of radiation education?” Over 89.0% of the participants responded positively.

## 5. Conclusions

In this study, knowledge among the general public and medical professionals in Oman on the topic of radiation and nuclear applications was investigated. We primarily aimed to measure the public’s knowledge and awareness in this field. Additionally, we assessed the knowledge of medical professionals in additional areas in the field. A total of 500 participants took part in the survey, 75 of whom were medical professionals. The outcomes and findings from the survey demonstrate that measures need to be taken to educate the public and medical professionals in the field of ionizing radiation and nuclear applications in general. Knowledge among the public and medical professionals was found to be intermediate and inadequate on introductory fundamental topics, namely, the nature of radiation, the origin of radiation in nature, radiation protection, and radiation applications. Recommendations include providing comprehensive and integrated courses and training programmes in this field through the education system in Oman. Anticipated outcomes include an accurate and controlled source of information and higher levels of knowledge in the field of radiation and nuclear energy among both the public and medical professionals in Oman.

## Data Availability

Data are available upon justified request.

## Appendix A. Survey Text

### Radiation Awareness Survey

The study seeks to assess the general public’s knowledge in Oman on radiation and nuclear energy. Your valuable contribution to this research is highly appreciated.

(* Indicates required question).

1. Demographic Information
  1.1. Age group *
    ○ 18 years old or less
    ○ 18–24 years old
    ○ 25–34 years old
    ○ 35–44 years old
    ○ 45–54 years old
    ○ 55–64 years old
    ○ 65 years old or older
  1.2. Gender *
    ○ Male
    ○ Female
    ○ I don’t wish to declare
  1.3. Education *
    ○ Less than high school degree
    ○ Diploma or high diploma
    ○ Bachelor’s degree
    ○ Graduate degree (e.g., Master’s, PhD, M.D)
2. Basic Knowledge in Radiation *
  2.1. Do you think radiation exists? *
    ○ Yes
    ○ No
  2.2. Radiation can be seen and touched *
    ○ Yes
    ○ No
    ○ I don’t know
  2.3. Radiation is a form of energy. *
    ○ Yes
    ○ No
    ○ I don’t know
  2.4. Radiation is one type only. *
    ○ Yes
    ○ No
    ○ I don’t know
  2.5. Humans and living creatures can receive radiation externally only. *
    ○ Yes
    ○ No
    ○ I don’t know
  2.6. Radiation is always harmful and can’t be useful. *
    ○ Yes
    ○ No
    ○ I don’t know
  2.7. Can radiation be contagious? *
    ○ Yes
    ○ No
    ○ I don’t know
  2.8. Naturally occurring radioactive material, also known as NORM, can be found anywhere around the world? *
    ○ Yes
    ○ No
    ○ I don’t know
  2.9. Generally, we receive radiation in our everyday life. *
    ○ Yes
    ○ No
    ○ I don’t know
3. Background Knowledge in Radiation
  3.1. Do you have prior knowledge in radiation science. *
    ○ Yes
    ○ No
  3.2. How familiar are you with the concept of radiation? (scale from 1 to 5) *
    5 very familiar, 4 familiar, 3 average familiarity, 2 not very familiar, 1 not familiar at all
  3.3. Which of the following types of radiation are you aware of? (Select all that apply) *
    ○ Alpha particles
    ○ Beta particles
    ○ Gamma rays
    ○ X-rays
    ○ Neutron radiation
    ○ None of the above
  3.4. Where does natural radiation come from? (Select all that apply) *
    ○ Cosmic rays
    ○ Radon gas
    ○ Soil and rocks
    ○ Medical X-rays
    ○ Nuclear power plants
  3.5. What are the common uses of radiation in medicine? (Select all that apply) *
    ○ X-ray imaging
    ○ CT scans
    ○ Radiation therapy for cancer
    ○ MRI scans
    ○ Ultrasound imaging
  3.6. Which of the following is a common safety measure when working with radiation? *
    ○ Wearing lead aprons
    ○ Using shielding materials
    ○ Limiting exposure time
    ○ Keep a distance from radiation sources
    ○ All of the above
  3.7. Can high levels of radiation be harmful to health? *
    ○ Yes
    ○ No
    ○ Not sure
  3.8. Have you ever been concerned with radiation exposure? *
    ○ Yes
    ○ No
    ○ Not sure
  3.9. Where do you usually get information about radiation and its effects from? * (Select all that apply)
    ○ News media
    ○ Educational programs/institutions
    ○ Healthcare providers
    ○ Online resources
    ○ Government agencies
    ○ Social media
  3.10. Would you be interested in learning more about radiation safety and its effects? *
    ○ Yes
    ○ No
    ○ Maybe
  3.11. Do you think radiation education is important? *
    ○ Yes
    ○ No
    ○ Maybe
  3.12. Do you think universities need to promote the correct awareness of radiation knowledge? *
    ○ Yes
    ○ No
    ○ Maybe
4. Medical Professionals (General Knowledge on Radiation)
  4.1. Do you work in the medical field? *
    ○ Yes
    ○ No
  4.2. Radiation can cause harmful effects. *
    ○ True
    ○ False
    ○ I don’t know
  4.3. X-rays used in medical imaging can cause more harm than benefit. *
    ○ True
    ○ False
    ○ I don’t know
  4.4. The human body will be radioactive after undergoing an X-ray examination. *
    ○ True
    ○ False
    ○ I don’t know
  4.5. Both computer tomography (CT) and magnetic resonance imaging (MRI) use ionising radiation for their examination. *
    ○ True
    ○ False
    ○ I don’t know
  4.6. Cancer will definitely occur after radiation exposure. *
    ○ True
    ○ False
    ○ I don’t know
  4.7. Medical professionals need protection while using equipment that utilises radiation. *
    ○ True
    ○ False
    ○ I don’t know
  4.8. Medical professionals need more training in the topic of radiation education. *
    ○ True
    ○ False
    ○ I don’t know
